# Clinical research: low-level laser therapy in accelerating orthodontic tooth movement

**DOI:** 10.1186/s12903-021-01684-z

**Published:** 2021-06-28

**Authors:** Junyi Zheng, Kai Yang

**Affiliations:** Department of Orthodontics, School of Stomatology, Capital Medical University, No. 4, Tiantanxili, Beijing, China

**Keywords:** Low-level laser, Orthodontic tooth movement, IL-1β, RANKL, OPG

## Abstract

**Background:**

The present study aimed to investigate the effects of low-level laser therapy (LLLT) on orthodontic tooth movement and its correlation with the levels of interleukin-1β (IL-1β), receptor activator of nuclear factor kappa B ligand (RANKL) and osteoprotegerin (OPG) in gingival crevicular fluid (GCF).

**Methods:**

This split-mouth design study included 12 patients scheduled for the extraction of both upper first premolars. Patients were randomly selected for experimental group that received left- or right-side radiation with a diode laser (810 nm wavelength, 100 mW power output, 6.29 J/cm^2^ energy density). Laser treatment was applied on days 0, 7, 14, and 21, after loading the canine retraction forces. GCF concentrations of IL-1β, RANKL, and OPG were analyzed. The upper arch of each patient was scanned with an intraoral scanner to assess tooth movement.

**Results:**

The cumulative tooth movement over 28 days was significantly higher in the laser group than in the control group. We observed significant reductions in OPG levels and increases in IL-1β and RANKL levels in GCF samples on the experimental sides.

**Conclusion:**

With the parameter settings used in this study, LLLT could, to some extent, lead to changes in bone metabolism, which could accelerate orthodontic tooth movement.

*Trial registration*: Chinese Clinical Trial Registry, ChiCTR2000039594. Registered 2 November 2020—Retrospectively registered, www.chictr.org.cn/edit.aspx?pid=62465&htm=4.

## Background

The essence of orthodontics is tooth movement in response to applied orthodontic forces. When deciding whether to undergo fixed orthodontic therapy, it is important for patients to avoid prolonged treatment periods, due to the increased risk of gingival inflammation and dental caries. Moreover, frequent visits can be inconvenient.

To date, many groups have attempted to find approaches for stimulating bone remodeling that could increase the rate of orthodontic tooth movement (OTM) including the local injection of drugs, physical stimuli, and corticotomy [1]. Injections and corticotomy are associated with unpredictable systemic effects, local pain, and discomfort which limit a wide application of these methods in clinical practice. Physical therapy such as low-level laser therapy (LLLT) has been considered a better option for abbreviating treatment time, due to the minimal invasiveness and safety. The energy output of LLLT is sufficiently low to avoid temperatures above 36.5℃, or normal body temperature [2].

Accelerating tooth movement with LLLT has been the cynosure of recent studies. Most previous animal and human studies have shown that laser irradiation could significantly accelerate tooth movement [3–6]. In contrast, some studies reported that LLLT had no effect on the rate of OTM [7, 8]. In addition, few studies have focused on the response of bone remodeling factors to orthodontic force in conjunction with LLLT.

The present study aimed to evaluate the effects of LLLT on the retraction of human maxillary canines. We also aimed to assess the accompanying changes in the levels of receptor activator of nuclear factor kappa B ligand (RANKL), osteoprotegerin (OPG), and interleukin-1β (IL-1β) in gingival crevicular fluid (GCF). Assessing these bone remodeling mediators in a sterile inflammatory process induced by orthodontic force might improve our understanding of the molecular mechanisms that underlie LLLT-induced accelerated tooth movement.

## Methods

### Human subjects

The study population comprised 12 subjects (four men and eight women), aged 18 to 28 years. All patients had a clinical indication for extracting both upper first premolars and distalizing the bilateral maxillary canines. All patients were instructed to maintain good oral hygiene practices over the entire duration of the study.

Patients were selected according to the following criteria:

1. Adequate nutrition, with no sign of systemic illness, pregnancy, or lactation.
2. No long-term medical treatment that could interfere with bone metabolism, such as analgesics and nonsteroidal anti-inflammatory medicine.
3. No previous orthodontic treatment.
4. Good oral hygiene, no gingival recession, no radiological evidence of periodontal bone loss, a probing depth < 3 mm, and a gingival index < 1.

Each patient was informed about the procedures, and all signed informed consent forms. Ethical approval was obtained from the Research Ethics Committee of Hospital. No patients were lost to follow-up during the clinical trial.

### Experimental design

This was a split-mouth study, where the same materials and procedures, except laser irradiation, were applied to both groups. The left and right halves of the upper arcades were randomly assigned to the experimental (laser group) and control groups using coin toss method.

1. Orthodontic treatment

The orthodontic treatment was initiated 2 weeks after the maxillary first premolars were extracted. Preadjusted edgewise brackets with an MBT prescription (3 M Gemini brackets; 3 M Unitek, Monrovia, CA) with 0.022*0.028-in slots were used in this experiment. The canines were leveled and after that, a final working wire of 18*25-in stainless steel was placed. A transpalatal arch was cemented on both first molars to provide posterior anchorage. A nickel-titanium closed-coil spring was used to retract each canine. This instrument delivered a force of 150 g, measured with a dynamometer. After this point, a 28-day observation period was began.

(2) Low-level laser irradiation

The low-level laser device used in this study was a semiconductor diode laser (Doctor Smile Kombi, LAMBDA Spa, Italy). It was operated at a wavelength of 810 nm, a power output of 100 mW, and an energy density of 6.29 J/cm^2^, in continuous wave mode. The laser was equipped with a flexible fiber optic cable attached to a handpiece. The tip of the handpiece was held perpendicular and in direct contact with the mucosa. The laser irradiation was applied at 4 points (mesial buccal, distal buccal, mesial lingual, and distal lingual) for 40 s on each surface. LLLT was initiated at the beginning of retraction, and it was repeated on days 7, 14, and 21 by the same operator. During laser application, eye protection glasses, provided by the manufacturer, were worn by both the operator and the patient.

### Data collection

**Fig. 1:**
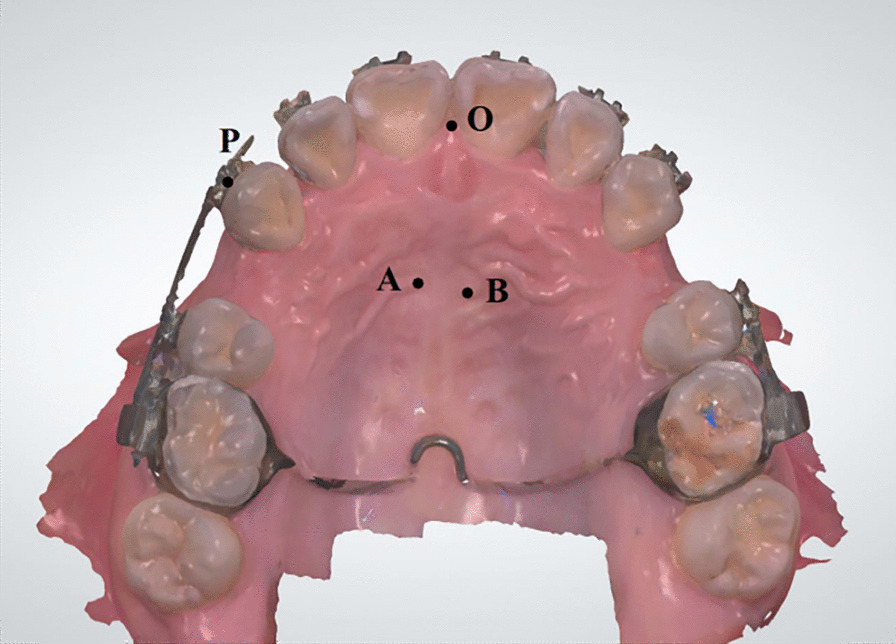
The left-hand coordinate system applied to image reconstructions of the mouth for measuring retraction distances. O, incisive papilla; A, median end of the right third palatal rugae; B, median end of the left third palatal rugae; P, midpoint of the orthodontic bracket on the occlusal edge. The XY plane was established based on points O, A, and B. Point O was regarded as the coordinate origin, and the X-axis points away from the origin in the O-A direction. The Y-axis is perpendicular to the X-axis through the origin, and the right side of the upper dental arch was in positive direction

To assess the amount of canine retraction, the upper arch of each patient was scanned with an intraoral scanner (3Shape A/S, Copenhagen, Denmark) on days 0, 7, 14, 21, and 28 (Fig. 1). Geomagic Studio software (Raindrop Geomagic, US) was used to reconstruct a 3D model, and a left-hand coordinate system was established, based on the incisive papilla and the median end of the bilateral third palatal rugae. The canine position was represented by the midpoint on the occlusal edge of the orthodontic bracket. The width of the canine bracket was used as the calibration standard. In the coordinate system, the distance (d1) between two points from the midpoint (M) on the mesial edge of the canine bracket to the midpoint (D) of the distal edge was calculated. We used the distance measuring tool to measure the actual distance (d2) between two points (M and D). Then, we defined the formula for the coefficients (n): n = d1/d2. The coordinates of canines at two time points were recorded and the retraction distances was calculated according to the following mathematical formula. The amount of canine retraction was measured three times and took the average.

$$d = \left\{ \sqrt {[(x1 - x2)^{2} + (y1 - y2)^{2} + (z1 - z2)^{2} ]}\right\} /n$$

Samples of gingival crevicular fluid (GCF) were collected from both the experimental and the control groups on days 0, 7, 14, 21, and 28. Briefly, paper points were inserted into the gingival sulcus of the canines at 4 sites (mesial buccal, distal buccal, mesial lingual, and distal lingual), until mild resistance was felt, and then the papers were left in place for 60 s. After GCF volume was measured, the paper points were transferred to Eppendorf tubes and stored at − 80 °C until the day of analysis within 1 month. The volume of GCF was calculated with the specific weight as 1 g/ mL. A certain volume of PBS buffer (0.01 mol/L, pH7.4) was added to each tube for dilution. Eppendorf tubes were shaken in a comfort mixer at room temperature for 40 min and the supernatant was extracted into new tubes after centrifugation. The concentrations of IL-1β, RANKL, and OPG in GCF were determined with the human IL-1β ELISA kit (R&D Systems, Minneapolis, MN, US), the human OPG ELISA kit (R&D Systems), and the human RANKL ELISA kit (R&D Systems), according to the manufacturer's instructions. The results were calculated using the standard curves set for each biomarker to determine the concentration of each sample. All samples and standards were assayed in duplicate. Data collections were done by the same operator.

### Statistical analysis

Statistical analyses were performed with SPSS 23.0. Values are expressed as the mean ± standard deviation (SD). Normality of the variables was ascertained by the K–S test in the control and experimental groups. Paired t-tests were used to compare the distance and velocity of canine movement between the experimental and control groups. Paired t-tests and one-way ANOVA were used to compare the levels of bone remodeling factors between and within the groups, respectively. *P < 0.05 was considered significant.

## Results

### Cumulative tooth movement

**Fig. 2:**
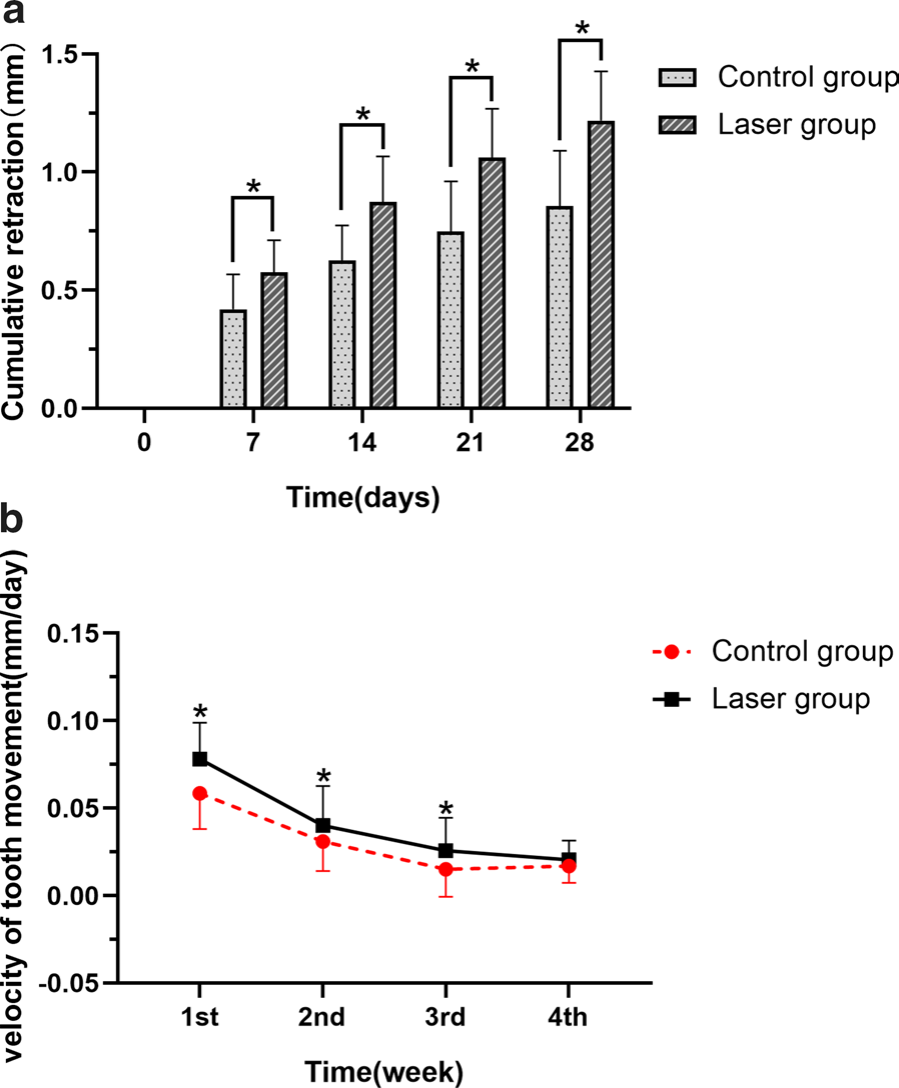
Comparison of movement and velocity with and without laser treatment. **a** The cumulative retraction of canines and **b** the velocity of tooth movement are shown in the control and laser groups throughout the study period. *P < 0.05 was considered significant, based on the Paired t test

Figure 2a displays the Cumulative distance achieved in both groups during the experimental period. At the end of 4 weeks of retraction, the canines were retracted 1.15 ± 0.29 mm on the laser side and 0.85 ± 0.23 mm on the control side. At all time points. the laser group showed significantly larger distalization than the control group. The mean retraction velocity was significantly greater in the laser group than in the control group (Fig. 2b), until the 4th week.

### Gingival crevicular fluid concentrations of IL-1β, RANKL, and OPG

**Fig. 3:**
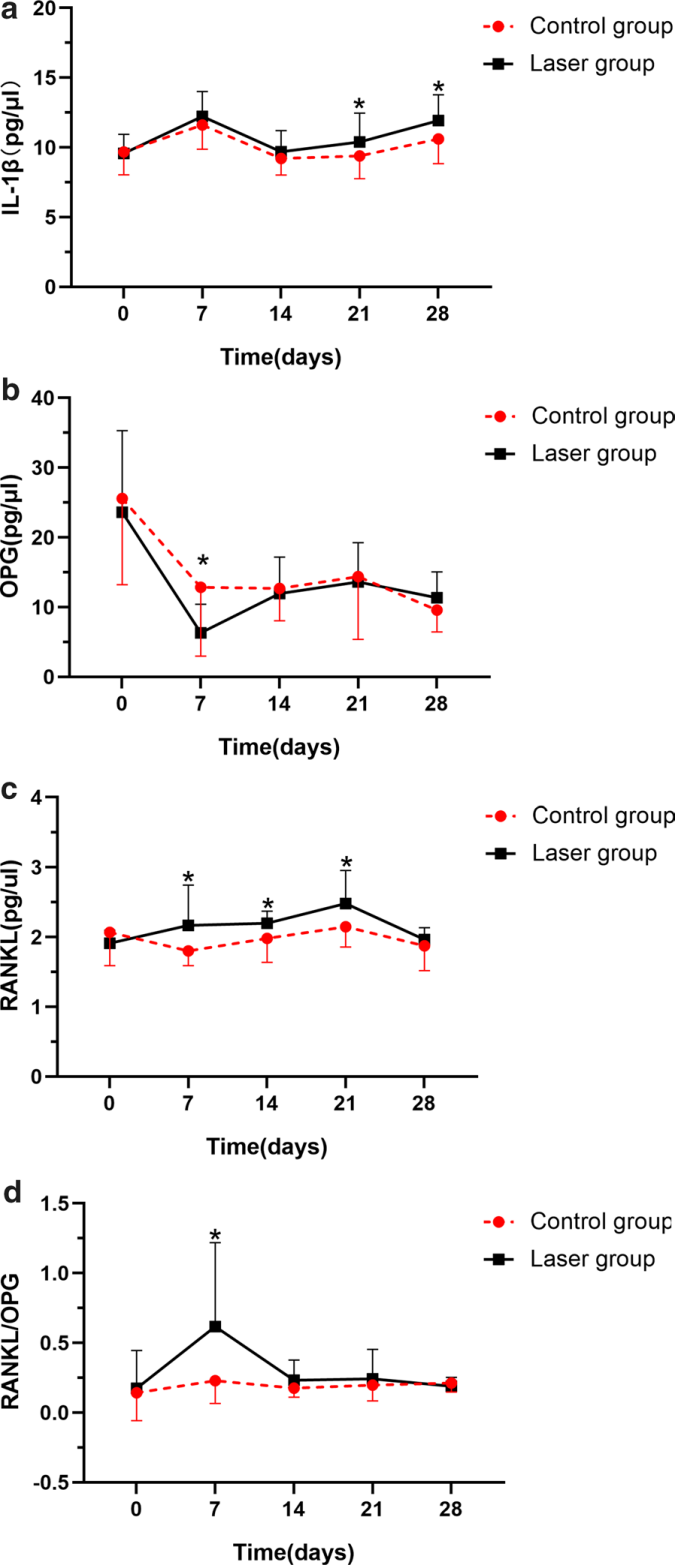
Changes in cytokine levels in the gingival crevicular fluid (GCF) throughout the study period. GCF concentrations of **a** IL-1β, **b** OPG, and **c** RANKL, and **d** the RANKL/OPG ratio are shown for control and laser groups. *P < 0.05 was considered significant, based on the Paired t test (for intergroup comparisons) or one-way ANOVA (for comparisons to baseline)

Table 1 shows the GCF levels of IL-1β, RANKL, and OPG and the RANKL/OPG ratios at all time points. GCF levels of IL-1β reached a maximum on day 7 in both groups. The change from baseline was significant on the 7th day. The laser group had significantly higher IL-1β levels compared to the control group on days 21 and 28 (Fig. 3a). After placing the orthodontic device for retraction, OPG expression was depressed at first, and then it slightly increased, but to a lower level than baseline in both groups. The minimum OPG value was observed on day 7 in the laser group and on day 14 in the control group. On day 7, the OPG concentrations were significantly lower in the laser group than in the control group (Fig. 3b). The RANKL level in the laser group was significantly higher than baseline on day 21; in contrast, RANKL levels remained practically constant in the control group. RANKL expression was significantly different on the laser side compared to the control side on days 7, 14, and 21 (Fig. 3c). In the laser group, the RANKL/OPG ratio increased, reached a maximum on the 7th day, and then returned to baseline (Fig. 3d). In the control group, the RANKL/OPG ratio was not significantly different from baseline during the entire experiment. The laser group ratio was significantly different from the control and baseline ratios only on day 7.

**Table 1:** Levels of GCF cytokine concentrations in the control and laser groups during the study period

Cytokine	Day	Control group		Laser group		Difference between groups (P value)
		Mean ± SD	Change from baseline (P value)	Mean ± SD	Change from baseline (P value)	
IL-1β (pg/μl)	0	9.67 ± 1.63		9.581 ± 1.36		0.585
	7	11.61 ± 1.73	0.005*	12.22 ± 1.80	0.000*	0.092
	14	9.21 ± 1.19	0.482	9.69 ± 1.51	0.874	0.317
	21	9.39 ± 1.63	0.665	10.39 ± 2.07	0.260	0.001*
	28	10.60 ± 1.76	0.161	11.93 ± 1.84	0.002*	0.013*
OPG (pg/μl)	0	25.58 ± 12.36		23.63 ± 11.68		0.076
	7	12.84 ± 9.86	0.001*	6.30 ± 4.13	0.000*	0.008*
	14	12.69 ± 4.65	0.000*	11.97 ± 5.21	0.000*	0.499
	21	14.38 ± 8.99	0.002*	13.63 ± 5.63	0.001*	0.686
	28	9.58 ± 3.12	0.000*	11.36 ± 3.68	0.000*	0.066
RANKL (pg/μl)	0	2.07 ± 0.48		1.91 ± 0.19		0.207
	7	1.80 ± 0.21	0.430	2.17 ± 0.58	0.088	0.039*
	14	1.98 ± 0.34	0.833	2.20 ± 0.17	0.058	0.015*
	21	2.15 ± 0.29	0.844	2.48 ± 0.47	0.000*	0.031*
	28	1.88 ± 0.36	0.638	1.97 ± 0.17	0.710	0.253
RANKL/OPG	0	0.14 ± 0.20		0.17 ± 0.27		0.187
	7	0.23 ± 0.16	0.118	0.62 ± 0.60	0.001*	0.032*
	14	0.18 ± 0.64	0.557	0.23 ± 0.17	0.655	0.113
	21	0.20 ± 0.11	0.321	0.24 ± 0.21	0.596	0.343
	28	0.21 ± 0.07	0.203	0.19 ± 0.06	0.901	0.284

*P < 0.05 was considered significant, based on the Paired t test (for intergroup comparisons) or one-way ANOVA (for comparisons to baseline)

## Discussion

### The effects of LLLT on OTM

Biological, OTM can be defined as the remodeling of periodontal tissues, particularly those surrounding alveolar bone, in response to mechanical force. Cytological studies have evaluated low-level laser (LLL) effects on various cells that are closely related to bone remodeling, particularly osteoblasts [9]. Those studies showed that LLLT could promote proliferation and differentiation. Animal studies have suggested that LLLT could positively impact bone regeneration and accelerate the velocity of experimental tooth movement [3, 4].

A number of clinical trials have evaluated the effects of LLLT over time during orthodontic treatments. Cruz et al. conducted a self-control test and found that, after LLL irradiation (780 nm, 20 mW, 5 J/cm^2^), canine retraction was significantly accelerated [5]. The velocity over 60 days was 34% faster in the laser group than in the control group. In another study, Youssef et al. showed that, with laser-irradiation (809 nm, 100 mW), orthodontic movement was 98% faster than without radiation, till the end of the retraction phase [6]. Similar to those reports, we found that the lased side had 35% faster retraction velocity over a period of 4 weeks compared to the control side. This finding might have resulted from the biostimulation promoted by LLLT. In addition, the rate of retraction reached a peak in the 1st week, but then it slowed down gradually in both groups. This finding suggested that LLLT did not change the pattern of tooth movement caused by orthodontic force.

However, Marquezan et al. found that LLLT failed to accelerate OTM when they applied an 830 nm laser with a high energy density of 6000 J/cm^2^ in rats [7]. Limpanichkul et al. applied an 860 nm diode laser on the canines with a dose of 25 J/cm^2^ and concluded that LLLT had no effect on the rate of OTM [8]. The controversial results observed among studies could probably be ascribed to the different experimental protocols, including differences in the laser wavelength, output power, irradiation time, treatment interval, and so on. In addition to the parameters related to the tissues subjected to laser therapy, wavelength is an important factor associated with the effect of LLLT. At present, it is widely accepted that the optical window for biostimulation is in the wavelength range of 550–950 nm approximately, where laser transmission is nearly largest. Moreover, infrared radiation has a high penetration depth in tissue, due to its low absorption coefficient in hemoglobin and water [5]. Another important factor that affect the therapeutic effect of the laser is the dosage. According to previous studies, lasers with energy densities in the range of 2 to 12 J/cm^2^ are capable of inducing biostimulation [10]. So, when the diode laser had a wavelength of 810 nm and an energy density of 6.29 J/cm^2^ in our study, we observed that the rate of OTM increased.

In addition, there may be dose response differences between humans and animal models and some studies lacked descriptions of important details relevant to the study designs. Thus, a direct comparison among various studies was a relatively arduous task. Although different LLLT parameters were shown to be effective for accelerating OTM in humans, more studies are required to determine the optimal LLLT settings.

### The accompanying changes in the levels of bone remodeling mediators

During orthodontic treatment, the transduction of mechanical forces to the cells triggers biological and biochemical responses. The early stage is characterized by an aseptic inflammation that ultimately promotes adaptive periodontium remodeling. Bone resorption is considered a rate-limiting step in remodeling. Thus, in this context, the velocity of OTM depends on the recruitment and differentiation of mature osteoclasts and precursors and the number of functional cells at the bone-periodontal ligament interface [11].

IL-1β is a pro-inflammatory cytokine secreted by various cells in the immediate response to mechanical stress. IL-1β was shown to be the earliest identifiable marker involved in bone resorption, with its specific capacity to stimulate osteoclast survival, differentiation, and function. Numerous studies have demonstrated that IL-1β levels were up-regulated after force application, and then declined over time [12, 13].

Similarly, in the present study, we observed that IL-1β levels were significantly elevated, and the concentration peaked on day 7. After that, the levels declined to near baseline. At all observation time points, higher levels of IL-1β were detected on the lased side compared to the control side, but these intergroup differences were not significant until day 21. This observation suggested that a biological reaction might have occurred in response to a stimulus other than orthodontic force. Consequently, we speculated that the significantly higher levels of IL-1β observed in the laser group were attributable to an induction by LLLT, and this effect gradually appeared, as the number of exposures increased. Similar findings were reported by Varella et al., who found that the mean concentration of IL-1β in the experimental group was 2.5-fold higher in the 1st month and fourfold higher in the 2nd month, compared to the levels observed in the control group [14]. Longer observation times are required to determine whether IL-1β levels might continue to increase as a consequence of the biostimulation effect induced by LLLT.

It is increasingly evident that RANKL exerts a pivotal role in osteoclastogenesis. When RANKL binds to its specific receptor (RANK), it initiates intracellular signaling cascades that result in bone resorption. This binding interaction is inhibited by a soluble decoy receptor, called OPG. In most previous clinical studies, OPG levels tended to decrease or remained constant, and RANKL levels tended to increase during the early stage of OTM [15–17].

In the present study, both groups displayed significant reductions in OPG levels, then slight elevations, but the levels remained low relative to baseline. This finding was consistent with the findings of Toygar et al., who found that OPG concentrations were significantly downregulated during the first hour, and they remained low throughout the 3-month observation period, compared to baseline measurements [17]. Different studies have reported conflicting results regarding the expression of RANKL. Several studies found significant increases at 24 h [15, 16], 48 h [18], or on day 42 [19]; in contrast, our results showed that RANKL levels remained relatively constant in the control group. This discrepancy might be explained by the variability in study designs, follow-up intervals, or experimental periods. We observed significantly lower OPG values on day 7 in the laser group compared to the controls. The low OPG values contributed to the high RANKL/OPG ratio, which was correlated to the higher velocity of OTM in the laser group compared to controls. This might be explained by the widely accepted view that initial phase of orthodontic tooth movement is either with rapid displacement of the tooth in the periodontal ligament space or bending of the alveolar bone [18]. In addition, high RANKL/OPG ratio was likely to reveal high level of the differentiation and activity of osteoclasts. On the other hand, we also observed significant increases in RANKL levels in the LLLT group compared to baseline on day 21 and compared to the control group on days 7, 14, and 21. These significant intragroup and intergroup differences might have resulted from a biostimulation effect induced by LLLT. Consistent with these findings, Domínguez et al. reported that, although they observed no significant differences in the GCF concentrations of RANKL and OPG or their ratio between the laser and placebo sides during the experimental period, they detected somewhat higher RANKL/OPG ratios in the laser group, compared to the control group [18]. That finding indicated that bone metabolism was affected to some extent by the application of orthodontic force combined with laser irradiation.

## Conclusion

In the conditions of this present randomized controlled trial, we concluded that LLLT could have clinical utility in accelerating OTM, due to its biostimulatory effects, which elicited an enhanced biological response in the periodontium adjacent to the tooth. More studies are needed to investigate different irradiation parameters, longer experimental periods, and more frequent time points to explain the mechanisms underlying the biostimulation effects, to find optimal laser settings, and to reveal possible side effects.

## Data Availability

The datasets analysed during the current study are available from the corresponding author on reasonable request.

## Abbreviations

LLLT: Low-level laser therapy
IL-1β: Interleukin-1β
RANKL: Receptor activator of nuclear factor kappa B ligand
OPG: Osteoprotegerin
GCF: Gingival crevicular fluid
OTM: Orthodontic tooth movement
LLL: Low-level laser
